# The climate change problem: promoting motivation for change when the map is not the territory

**DOI:** 10.3389/fpsyg.2015.00131

**Published:** 2015-02-11

**Authors:** Idit Shalev

**Affiliations:** Department of Education, Zlotowski Center of Neuroscience, Ben-Gurion University of the NegevBeer Sheva, Israel

**Keywords:** climate change, automatic processes, energy, goal pursuit, motivation for change, strategic intervention

The climate-change risks that have emerged in the wake of mass industrialization indicate the need for a transition to sustainable energy, but attempts to encourage people to adopt pro-environmental behavior often achieve only limited success (Whitmarsh and O'Neill, 2010; Chu and Majumdar, 2012). Indeed, despite the wealth of evidence that an energy transition is critical to our very survival, people in general are reluctant to change their energy related decisions and behaviors. I suggest that the key to understanding our stunted ability to address the problem of climate change is rooted in human motivation, which is defined as the process that moves individuals to action. Motivational barriers, which imply difficulties in energy, direction and action initiation, can be illustrated by the analogy of a car, which requires fuel as its energy source and a steering system to provide direction; without both, it cannot move (Deci and Ryan, 1985). Similarly, motivational processes are impelled by two modes of action: one, automatic, effortless and involving no conscious awareness, is responsible for people's immediate responses to the environment. The second is voluntary and conscious, and it mainly helps people navigate and plan for the future direction (Bargh, 1997; Baumeister and Bargh, 2014). From this perspective, the phenomenon of motivation for change is influenced by both implicit and explicit processes and determined by the energy required to navigate in a new direction and the extent to which resistance to change can be overcome (Prochaska and DiClemente, 1986). In what follows, I will describe the unique challenges associated with motivation for change in terms of the two modes of goal pursuit, first by examining the conscious, explicit difficulties associated with making climate change goal and implementing the corresponding means and then by describing the effects of automatic, implicit processes on immediate goal pursuit. Finally, based on social psychology perspective of motivation and strategic principles of psychological intervention, I will provide several recommendations for promoting the energy transition.

## Explicit goal pursuit

A clear sense of direction, essential to implement the behavioral change needed to promote an energy transition, is at the core of the ability to govern or to direct attention, resources, or action toward the realization of a particular goal (Higgins, 1989). Insofar as it is defined as a cognitive representation of a desired end-point that affects evaluations, emotions, and behaviors (Fishbach and Ferguson, 2007), a goal entails information about desired end-states that serve as reference points toward which behavior is directed (Carver and Scheier, 1981; Kruglanski et al., 2002; Shah et al., 2002).

The climate change problem, however, is exceptionally amorphous, providing no clear direction for goal pursuit in the quest for solutions. Therefore, much of the public discourse about climate change comprises critical evaluations of alternative goals or means to decide which should be pursued to achieve the aims of climate change control. Ample research in the field of social psychology suggests that chronic involvement in assessment and evaluation as opposed to movement from state to state is associated with paralysis and inaction (Kruglanski et al., 2000, 2010; Shalev and Sulkowski, 2009). Similarly, research has demonstrated that perceived threat inhibits one's readiness to have new experiences and to try unfamiliar directions. Individuals who feel they are under threat, therefore, tend to neglect their long-term, future planning goals in favor of the short-term goal of self-defense. Because the presence of threat stimulates the motive to reaffirm rather than to change the self, individuals typically resist the challenge entailed in altering their habitual behavior (Steele, 1988; Cohen and Sherman, 2014). As such, the presence of threat reduces individual readiness to modify judgment in the light of new evidence (Kruglanski, 1989). Likewise, the greater the ambiguity of the situation, the greater the need for a rapid answer (Kruglanski and Webster, 1996; Kruglanski, 2004), which breeds resistance to change as revealed in the real-world proclivity for political conservatism among many decision makers (Lewin, 1951; Jost et al., 2003).

Extending this line of thought, the pursuit of solutions to the climate change problem has motivational consequences for one's energy estimation and economy of action. Insofar as energy for goal pursuit is constrained by limited attentional resources (Kruglanski et al., 2002), the availability of energy to invest in behavior guided by a volitional change of habit is limited. Theories on goal-gradients suggest that short-term goals may undermine long-term goals because the strength of goal activation increases as a function of physical (e.g., Hull, 1932) or temporal proximity to the goal (Markman and Brendl, 2000). Therefore, investing energy in a distant goal is perceived as more costly, which helps explain why individuals prefer to conserve their energy for causes perceived as essential.

## Implicit goal pursuit

The climate change problem lacks important defining features such as geographic location or deadline. For example, climate deterioration has been defined using multiple terms (e.g., global warming, climate crisis, or climate change) interchangeably, indicating the absence of a clear direction in public discourse (Schuldt et al., 2011). Research has shown that given this uncertainty, individual orientation vis-à-vis global warming may be shaped by an alternative, automatic route to goal pursuit that functions without involving one's conscious awareness. Under such a scenario, external environmental stimuli trigger relevant mental representations that effect immediate action, but people are not, and usually remain, unaware of the influence exerted by those stimuli (Bargh et al., 2010; Loersch and Payne, 2011; Bargh et al., 2012). For example, the exposure to heat-related primes and to anchors for future rises in temperature increased the level of belief in climate change and the willingness to pay to combat global warming (Joireman et al., 2010; Risen and Critcher, 2011). The automatic mode of goal pursuit suggests that activation caused by physical experiences automatically spreads to their associated mental representations and may subsequently influence climate change decisions and behaviors (Bargh and Shalev, 2012; Meier et al., 2012). Likewise, there is evidence that estimated energy for action was automatically influenced by physical cues of thirst and dryness such that individuals reported greater fatigue and lower vitality, increased thirst, and decreased persistence in task completion. Taken together, these results suggest that the perception of somatic state automatically affects goal pursuit and may implicitly affects our motivation to change habitual behavior (Shalev, 2014).

## Can we influence the energy transition?

Thus far, we have examined the explicit and implicit motivational difficulties entailed in making decisions about the climate change problem and then in implementing the appropriate behavioral changes to address the problem. In his seminal work on the ecology of mind, Bateson (1972) argued that “the map is not the territory,” suggesting that the potential usefulness of future script is not a matter of its literal truthfulness, but rather, its utility relies on the extent to which its structure is similar to that of the territory itself. Because of the non-linear, infinite nature of cybernetics, a volitional plan could only be a partial representation of reality. Thus, goals should be close to the surface and structured within a flexible system that enables continuous change. From this theoretical perspective, the strategic approach of systemic psychological intervention is guided by a basic assumption that resolution of the problem does not require that it be fully understood. The general idea is that the solution serves as a “skeleton key” that can be used to unlock multiple problems (de Shazer, 1988, 1994). Based on this perspective, while the proposed solution may not match the problem exactly, the solution should be small enough that the system will accommodate the solution, which will move the system in a slightly different but preferable direction (de Shazer, 1994; Shoham et al., 1995).

Following this view and despite ambiguous end-states, policy makers should invest more energy in encouraging behavioral change that promotes more sustainable resource use than in engaging in the chronic evaluation of possible future end states. One strategy to increase energy for change is to exploit positive message framing to provide incentives for action initiation (Tversky and Kahneman, 1981; Custers and Aarts, 2005). Likewise, research findings indicate that gain-framed messages are more reassuring than loss-framed messages, and therefore, the former will help reduce the stress generated by ambiguous conditions and increase the willingness to adopt new behavioral directions (Rothman and Salovey, 1997). Based on the logic that expected incentive increases motivation for change, communication and regulations (e.g., carbon pollution standards) should be framed in terms of benefits rather than costs.

At the individual level, environmental policies should use external cues as sources of automatic action generation to increase the strength of the association between person and environment (Shalev and Bargh, 2011). Despite evidence that a positive attitude toward environmental protection does not align closely with one's behavior (e.g., Bamberg and Möser, 2007; Nigbur et al., 2010), the exposure to pro-environmental messages and the offering of incentives to use products that are in line with an energy transition may elicit more positive automatic evaluations and subsequently promote the consumer choices of individuals in their daily lives.

To strengthen the direction of action initiation, domestic policy should be occupied more with goal striving than with planning and anticipation (Gollwitzer, 1996). Because of the highly controversial nature of the climate change problem, policy should be designed inclusively by going beyond the immediate issue of problem resolution. For example, the goal of promoting healthy human-nature interaction by improving people's lifestyles could be associated with the corresponding means of attainment and with sub-goals in multiple domains (e.g., environment, health, economy, education, and science). The result is a goal system, associated with each other such that the pursuit of the energy transition goal will be perceived as complementary to the pursuit of other agree upon goals. Such a system of goals comprises incentives, increases action initiation and contributes to the immediate greater good of individuals and firms (e.g., subsidizing conservation technology and hybrid cars; encouraging research of new technologies). Similarly, the use of multifinal means (Kruglanski et al., 2013) may, in addition to the goal of energy transition, help attain other, less controversial or more inclusive goals, in so doing reducing the resistance for change.

Based on this reasoning, to increase motivation for change, policy efforts should create incentives for firms and individuals to pursue the most cost effective options for combatting climate change over time among all sectors, across national borders, and in the face of significant uncertainty. Well-designed national greenhouse gas mitigation policies can serve as the foundation for global efforts and as an example for emerging and developing countries. Following this view, international policy should link climate change initiatives with other contemporary values and interests (e.g., support in developing countries, international commerce, and import regulation). Likewise, cost certainty can be implemented as insurance against sudden, unexpected expenses and to reduce ambiguity.

## Conclusions

This paper presents a motivational perspective to the climate change problem, in the process showing the relevance of explicit and implicit processes in the decision-making and behavioral implementation phases. The approach was based on the assumption that the ambiguity of the condition entails an inherent absence of defining features that can be used to guide our decisions about how to approach the problem. As a result of such ambiguity, people typically concentrate their energy on risk assessment rather than on taking action. However, based on the reasoning that “the map is not the territory” and that the problem need not be fully understood to resolve it, strategies that promote behavioral change should be encouraged. These strategies, which may serve as “skeleton keys,” should also include positive message frames to increase people's energy for change, construct a system of goals that is more inclusive than the climate change problem and defined in terms of desired end states, provide incentives to promote energy transition behaviors, and increase the visibility of pro-environmental messages to generate more positive automatic evaluations that will result in environmentally friendly consumer choices.

### Conflict of interest statement

The author declares that the research was conducted in the absence of any commercial or financial relationships that could be construed as a potential conflict of interest.
